# Characterization and In Vitro Prebiotic Activity of Pterostilbene/β-Cyclodextrin Inclusion Complexes

**DOI:** 10.3390/molecules30061363

**Published:** 2025-03-18

**Authors:** Chuan-Chao Wu, Long Qian, Zhen Rong, Yu-Qi Li, Hui-Min Zhang, Rui-Yu He, Guo-Qiang Zhang

**Affiliations:** 1 College of Biology and Food Engineering, Anhui Polytechnic University, Wuhu 241000, China; 2309064@ahpu.edu.cn (C.-C.W.); 3220403211@stu.ahpu.edu.cn (L.Q.); 3220403214@stu.ahpu.edu.cn (Z.R.); 3220403213@stu.ahpu.edu.cn (Y.-Q.L.); zhm_catharine@ahpu.edu.cn (H.-M.Z.); 2 Wuhu Green Food Industrial Research Institute Co., Ltd., Wuhu 241000, China; 3 School of Biotechnology, Jiangnan University, Wuxi 214000, China

**Keywords:** pterostilbene, β-cyclodextrin, inclusion complexes, prebiotic, short chain fatty acids, gut microbiota

## Abstract

Pterostilbene (PTS) has multiple benefits, but poor water solubility and bioavailability limit its application. PTS/β-CD inclusion complexes were synthesized through the phase solubility method to enhance their water solubility. The inclusion complexes were characterized through Fourier transform infrared spectroscopy, scanning electron microscopy, X-ray diffraction, nuclear magnetic resonance, and molecular docking techniques. The results demonstrated that PTS and β-CD successfully created inclusion complexes with a host–guest ratio of 1:1 and a stability constant of 166.7 M^−1^. To further investigate its prebiotic function, simulated digestion experiments revealed that β-CD exhibited resistance to digestion, allowing it to reach the colon intact. During gastrointestinal digestion, PTS in the PTS/β-CD inclusion complexes was gradually released. Following digestion, the in vitro fermentation of healthy human feces further confirmed the probiotic properties. Compared to the β-CD and fructooligosaccharide (FOS) groups, the PTS/β-CD group significantly increased the production of acetic acid, butyric acid, and lactic acid, respectively. Additionally, beneficial bacteria, such as *Bifidobacterium* and *Lactobacillus*, proliferated in the PTS/β-CD group, while the relative abundance of potential pathogenic bacteria, such as *Lactococcus*, *Streptococcus*, and *Klebsiella*, was significantly reduced. Compared to the blank group, propionic acid and butyric acid concentrations in the β-CD group were significantly higher. The abundance of *Lactobacillus* and other key bacterial species in the β-CD group increased, while the relative abundance of *Klebsiella* and other pathogens decreased significantly. In conclusion, PTS/β-CD inclusion complexes altered the composition of intestinal flora, promoting the proliferation of beneficial bacteria and inhibiting the growth of harmful bacteria, thereby demonstrating dual probiotic functionality.

## 1. Introduction

**Figure 1:**
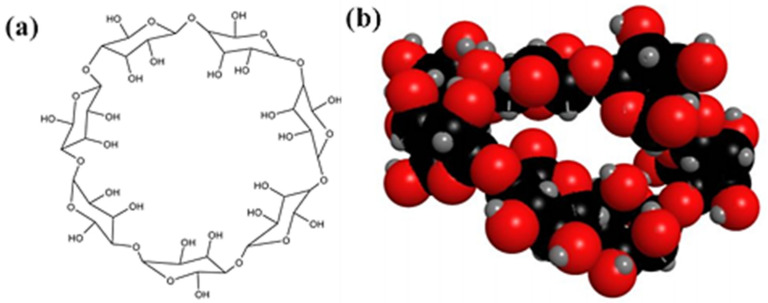
Molecular structure (**a**) and 3D model (**b**) of β-CD.

Cyclodextrins are cyclic oligosaccharides composed of glucopyranose subunits linked by α-1, 4-glucoside bonds [1,2]. Among various cyclodextrins, β-cyclodextrin (β-CD) is commonly utilized for encapsulate hydrophobic and volatile guest molecules, enhancing their solubility, stability, and bioavailability. This is due to its favorable water solubility (18.5 g/L), suitable cavity size (0.85 nm), and availability at a low cost (Figure 1). As a result, β-CD has broad application prospects in food, drug delivery systems, and medical fields [3,4,5,6].

**Figure 2:**
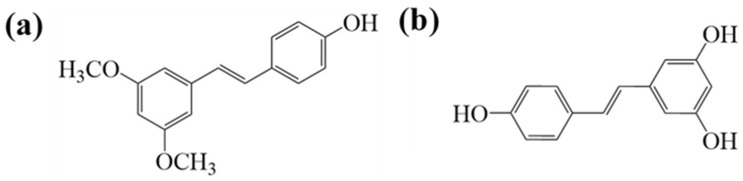
Chemical structure of pterostilbene (**a**) and resveratrol (**b**).

Pterostilbene (PTS) is a natural compound widely found in grapes and blueberries [7,8]. It has garnered significant attention for its various dietary benefits, particularly its role in promoting colon health [9]. However, PTS’s poor solubility in aqueous solutions (30 µg/mL) and low stability often result in degradation or metabolism in the digestive tract before it reaches the colon, which limits its commercial applications [10]. Therefore, improving the water solubility of PTS is crucial for enhancing its bioavailability. PTS contains two methoxy groups and one hydroxyl group in its structure. It is similar to resveratrol but has higher bioavailability than it. Therefore, it is regarded as the next generation of resveratrol (Figure 2) [11,12]. Thus, selecting a more suitable carrier is essential to enhance its solubility, protect it from harsh stomach conditions, and ensure its controlled release in the colon to modulate gut flora.

Research has indicated that gut microorganisms play a pivotal role in maintaining human health preserving the intestinal barrier’s integrity, enhancing immunity, inhibiting intestinal pathogens, and sustaining intestinal ecological balance [13]. Thus, preserving the diversity and dynamic balance of gut microbes is vital for overall health. Prebiotics are components that can be selectively utilized by beneficial microorganisms of the host and produce metabolites that benefit the host’s health [14]. Certain dietary fibers, particularly fructooligosaccharide (FOS), are widely recognized as ideal prebiotics [15]. Moreover, numerous studies have highlighted interactions between phenols and gut microbiota, suggesting that phenols could be promising prebiotic candidates. Phenols influence microbial diversity by selectively stimulating the growth or activity of beneficial bacteria in the colon, while inhibiting potentially pathogenic bacteria, yielding beneficial effects for the host [11,16]. In turn, gut microbes also modulate the activity of phenolic compounds. This reciprocal interaction influences the metabolism and bioavailability of phenols, converting them into beneficial metabolites [14]. After fermentation by gut microbes, the main metabolites of probiotics are short-chain fatty acids (SCFAs), which are crucial for sustaining the proper functioning of the large intestines as well as the structure and function of colon epithelial cells [13]. Based on this, we hypothesized that the combination of PTS with β-CD might be a key factor in PTS/β-CD’s health benefits. β-CD serves as a carrier for delivering PTS to the distal intestine, where microbial transformations could occur. However, the release of PTS from β-CD and its impact on the prebiotic properties of the PTS/β-CD inclusion complexes remain unclear.

Given the above, PTS/β-CD inclusion complexes were formulated utilizing the stirring solution method, and their stoichiometric ratio was measured by the phase solubility method. The PTS/β-CD were then characterized through diverse methods, including Fourier transform infrared spectroscopy (FT-IR), scanning electron microscopy (SEM), X-ray diffraction (XRD), nuclear magnetic resonance (NMR), and molecular docking. Additionally, the antioxidant properties and stability of the PTS/β-CD inclusion complexes were evaluated. Furthermore, their potential probiotic effects were investigated through in vitro simulated digestion and fecal fermentation using samples from healthy individuals.

## 2. Results and Discussion

### 2.1. Phase Solubility Method

**Figure 3:**
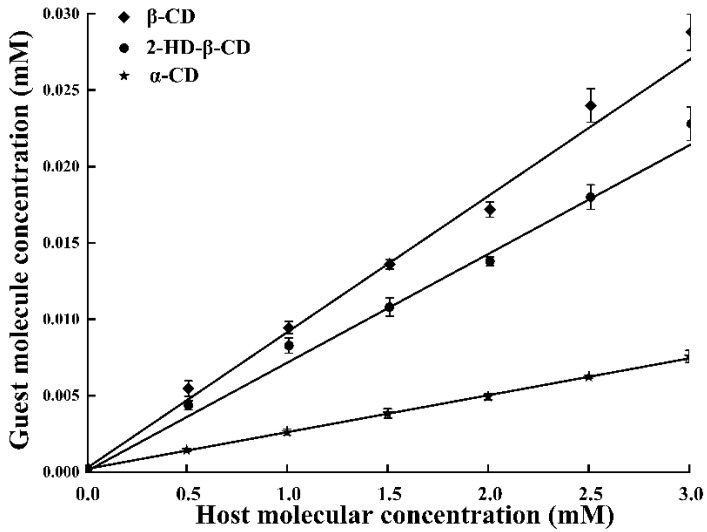
The phase solubility diagrams for PTS in the presence of host molecule at 25 ± 2 °C.

Based on the morphology of inclusion complexes by Higuchi et al. [17], the phase solubility curve between PTS and various host molecules displayed a typical A_L_-type curve, indicating a 1:1 molar ratio between β-CD and PTS (Figure 3), similar to the findings of Catenacci et al. [18]. Additionally, the solubility of PTS increased linearly with increasing host molecule contents (*R*^2^ > 0.99) [19]. The stability constant (*Kc*) typically ranged between 50 and 2000 M^−1^. If the *Kc* value was excessively high, the release of PTS from the host molecular cavity would be hindered. Conversely, low *Kc* values indicated that the host molecules were poorly suited as drug carriers [20]. The *Kc* values of β-CD, α-CD and 2-HD-β-CD inclusion complexes were 166.7, 132.8, and 54.9 M^−1^ (β-CD > 2-HD-β-CD > α-CD), respectively, indicating that the PTS/β-CD inclusion complexes were the most stable.

### 2.2. Characterization of PTS/β-CD Inclusion Complexes

#### 2.2.1. SEM Analysis

**Figure 4:**
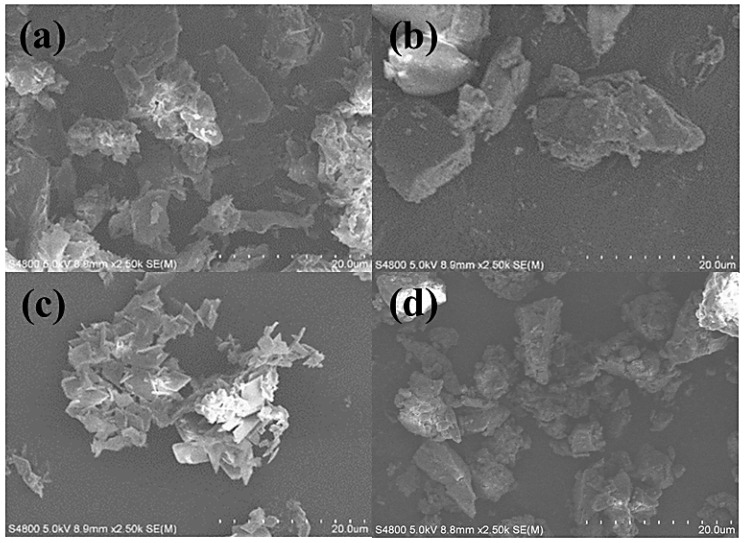
SEM images of β-CD (**a**), PTS (**b**), PTS/β-CD inclusion complexes (**c**), and PTS/β-CD physical mixtures (**d**).

In Figure 4a, β-CD appeared as an amorphous, irregular mass. At the same time, PTS took on an irregular shape (Figure 4b). The physical mixture of β-CD and PTS (Figure 4d) displayed an additional layer of fine particulate matter on top of β-CD, with crystalline clumps of PTS coexisting alongside the clumps of β-CD, indicating no interaction between the two components. However, in the PTS/β-CD inclusion complexes (Figure 4c), the basic structures of both PTS and β-CD were lost and replaced by lamellar formations. This transformation was likely due to the low crystallinity of both PTS and β-CD. Neither β-CD nor PTS retained their original forms. These results confirmed that β-CD and PTS successfully interacted to form PTS/β-CD inclusion complexes [21].

#### 2.2.2. FT-IR Analysis

**Figure 5:**
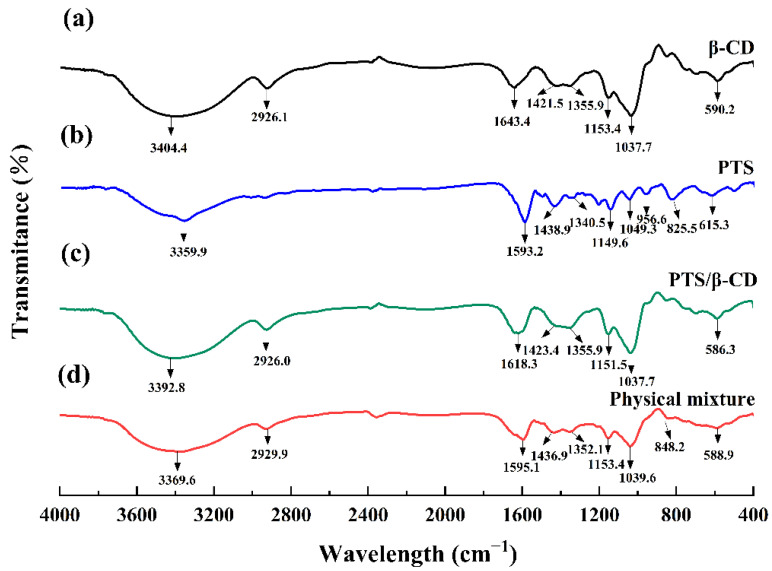
FT-IR patterns of β-CD (a), PTS (b), PTS/β-CD inclusion complexes (c), PTS/β-CD physical mixtures (d).

The FT-IR spectrum of β-CD revealed peaks at 3404.4 cm^−1^ and 2926.1 cm^−1^, corresponding to -OH stretching and C-H stretching vibrations, respectively. The 1643.4 cm^−1^ and 1037.7 cm^−1^ peaks were assigned to -OH bending and C-O stretching vibrations (Figure 5a) [18,22]. In the FT-IR spectrum of PTS (Figure 5b), typical absorption peaks appeared at 615.3, 825.5, 956.6, 1049.3, 1149.6, 1438.9, 1593.2, and 3359.9 cm^−1^ [23,24]. In the PTS/β-CD physical mixtures (Figure 5d), the characteristic peaks of both β-CD and PTS were present, showing weak or no interaction between the two compounds. However, in the PTS/β-CD inclusion complexes (Figure 5c), the absorption peaks of β-CD remained visible, while the peaks of PTS disappeared, suggesting that the inclusion of PTS within β-CD restricted its infrared vibrations. These results showed a rightward shift of the -OH bending vibration by 25 cm^−1^, and the -OH peak position shifted to a lower wavenumber compared to β-CD. This shift was caused by the interaction between the -OH group of PTS and the C-O-C group of β-CD, confirming the formation of the PTS/β-CD inclusion complexes [25].

#### 2.2.3. DSC Analysis

**Figure 6:**
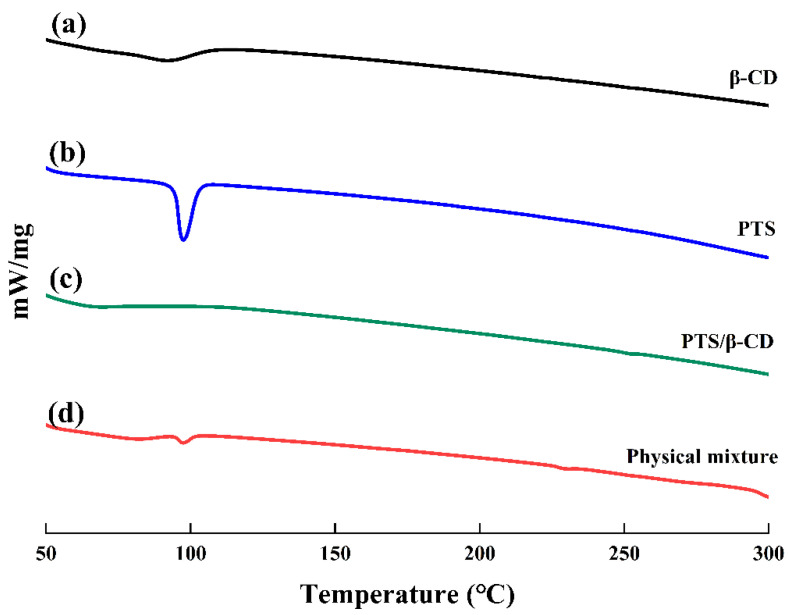
The DSC curves of β-CD (a), PTS (b), PTS/β-CD inclusion complexes (c), and PTS/β-CD physical mixtures (d).

β-CD exhibited a wide endothermic peak at 89 °C, attributable to the loss of moisture from β-CD (Figure 6a). It was found that PTS has a sharp melting peak at 97 °C, corresponding to its crystalline melting point (Figure 6b) [26]. However, the peak of β-CD at 89 °C shifted to 68 °C, and the intensity decreased, while the characteristic endothermic peak of PTS disappeared completely, which may be caused by drug molecules replacing water molecules in the β-CD cavity (Figure 6c). In contrast, the physical mixture represented a mere combination of the endothermic peaks observed for PTS and β-CD, indicating that the two substances were only a simple physical mixture without interacting with each other, and PTS still showed crystal characteristics in the physical mixture (Figure 6d) [27]. These results demonstrated that PTS existed in an amorphous form within the PTS/β-CD inclusion complexes, confirming that PTS was incorporated within the β-CD cavity to form the inclusion complexes.

#### 2.2.4. TG Analysis

**Figure 7:**
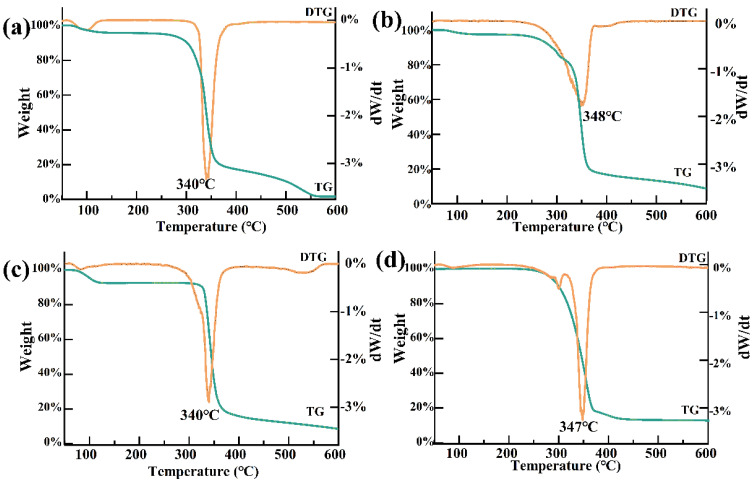
The TG curves of β-CD (**a**), PTS (**b**), PTS/β-CD inclusion complexes (**c**), and PTS/β-CD physical mixtures (**d**).

The β-CD weight loss curve showed two main stages. The first stage, occurring at around 100 °C, was primarily due to the loss of free and bound water from β-CD. The second stage, occurring between 300 and 400 °C, with a peak at around 340 °C, was associated with the degradation of β-CD (Figure 7a). For PTS, the mass loss occurred between 257 and 600 °C, with a weight loss rate of 91.16%, mainly due to the phase transition of PTS from liquid to gas (Figure 7b) [26]. After the establishment of the PTS/β-CD inclusion complexes, a gradual weight loss was observed between 300 and 400 °C, indicating improved thermal stability of PTS within the complex, which delayed its volatilization and release (Figure 7c). The trend of its weight loss curve corresponds with this research [18], but there were certain differences in the specific proportion of mass loss, which may be due to variations in experimental parameters, such as the heating rate and sample amount during the experiment. In contrast, the physical mixture showed weight loss at 300–400 °C due to the degradation of β-CD and volatilization of PTS, indicating no interaction between the two components (Figure 7d) [28]. These results demonstrated that PTS entering the hydrophobic cavity of β-CD disrupted its ordered structure, with PTS molecules encapsulated within the cavity, leading to the increased thermal stability of the host–guest complex.

#### 2.2.5. XRD Analysis

**Figure 8:**
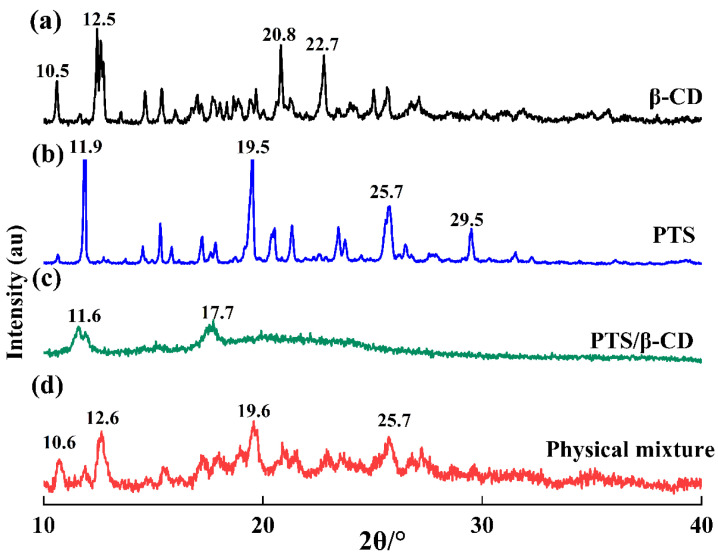
The powder XRD of β-CD (a), PTS (b), PTS/β-CD inclusion complexes (c), and PTS/β-CD physical mixtures (d).

XRD analysis represents a successful approach to verifying the formation of inclusion complexes [29]. When inclusion complexes form, guest molecules enter the β-CD cavity, leading to lattice changes and corresponding alterations in the diffraction pattern. The main diffraction peaks of β-CD (Figure 8a) were at 10.5°, 12.5°, 20.8°, and 22.7° (2θ), while the main diffraction peaks of PTS (Figure 8b) were at 11.9°, 19.5°, 25.7°, and 29.5° (2θ). These peaks showed several strong and sharp diffraction peaks, indicating an obvious crystalline structure. In the physical mixture, sharp diffraction peaks of both components were still visible, suggesting that the mixture was a simple combination of host and guest molecules without interaction (Figure 8d) [28]. In addition, due to prolonged grinding, the crystallinity of the physical mixtures was lower than that of the pure compound. However, in the PTS/β-CD inclusion complexes, these sharp diffraction peaks disappeared. Instead, two broad diffraction peaks appeared near 2θ values of 11.6° and 17.7°, confirming that, when PTS entered the β-CD cavity, it interacted with β-CD, resulting in reduced or lost crystallinity for both PTS and β-CD (Figure 8c) [30].

#### 2.2.6. NMR Analysis

**Figure 9:**
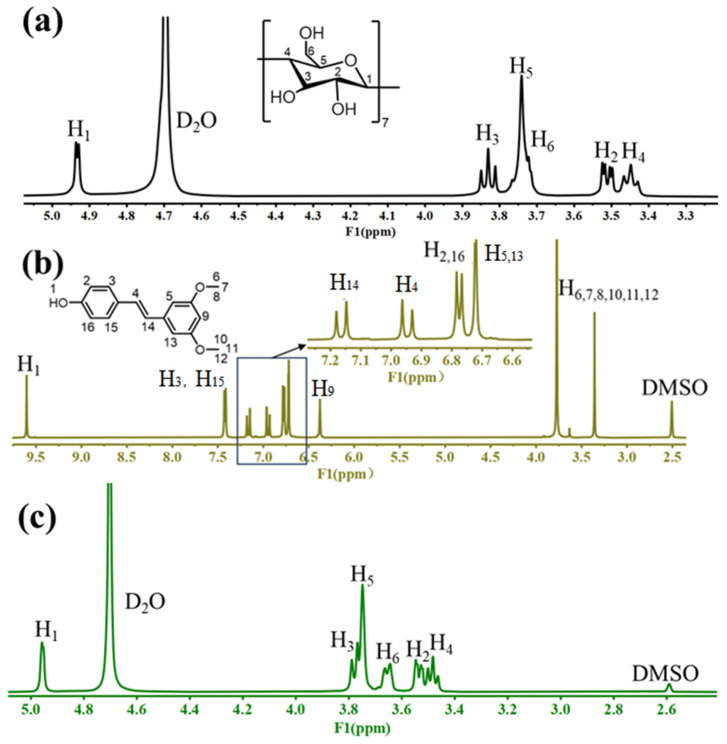
^1^H NMR spectra of β-CD (**a**), PTS (**b**), PTS/β-CD inclusion complexes (**c**).

The complexation mode can be deduced by comparing the chemical shifts of β-CD using ^1^H NMR before and after the inclusion process [28]. The proton signals of β-CD were detected at δ 4.93 (H_1_), δ 3.52 (H_2_), δ 3.83 (H_3_), δ 3.45 (H_4_), δ 3.76 (H_5_), and δ 3.72 (H_6_) ppm (Figure 9a). The proton signals of PTS were detected at δ 9.60 (H_1_), δ 6.78 (H_2_,_16_), δ 7.41 (H_3_,_15_), δ 7.15 (H_14_), δ 6.72 (H_5_,_13_), δ 3.76 (H_6_,_7_,_8_,_10_,_11_,_12_), and δ 6.95 (H_4_) ppm (Figure 9b) [31]. The formation of host–guest inclusion complexes altered the environment inside the β-CD cavity, causing up-field shifts in the H_3_ (Δδ = −0.09 ppm) and H_5_ (Δδ = −0.03 ppm) protons, positioned inside the hydrophobic interior of β-CD (Figure 9c). In contrast, the protons outside the cavity (H_1_, H_2_, and H_4_) showed no significant changes before and after inclusion [32]. The chemical shift of H_3_ was larger than that of H_5_, as H_3_ was located near the wide-mouth end of the cavity, while H_5_ was near the narrow-mouth end. This suggested that PTS was successfully situated within the β-CD hollow space, likely entering through the wide-mouth end near H_3_. After inclusion, the signal intensity of the characteristic peaks (PTS) was significantly weakened and overlapped with part of the signal of β-CD, further confirming the formation of molecular inclusion complexes between PTS and β-CD [26].

### 2.3. Molecular Docking Analysis

**Figure 10:**
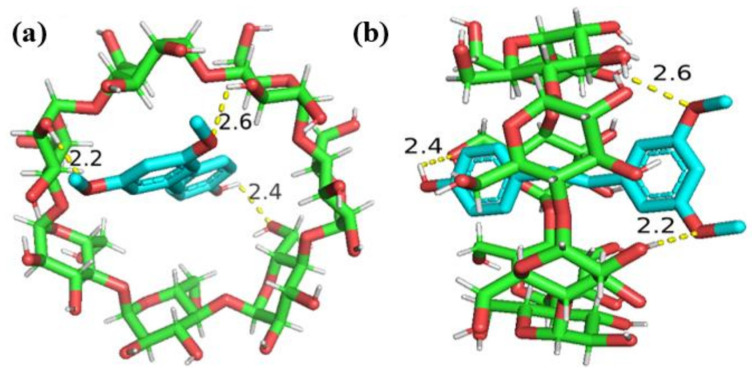
The minimum energy configuration of PTS/β-CD inclusion complexes. Top-view (**a**); Side-view (**b**).

The optimal conformation of the PTS/β-CD inclusion complexes was shown in Figure 10. PTS was able to completely enter the cavity of β-CD, with good spatial size matching, and the coordination inclusion stability was confirmed (the best binding energy was −6.7 kcal/mol). Additionally, the formation of hydrogen bonds further contributed to the inclusion complexes’ stability. The phenol hydroxyl group of PTS formed 2.4 Å hydrogen bonds with the oxygen atoms at the C_6_ position of β-CD, while the oxygen atoms of the two methoxy groups of PTS formed hydrogen bonds of 2.6 Å and 2.2 Å with the hydrogen atoms at the C_3_ position (C_3_-OH) of β-CD, respectively. This indicates that hydrogen bonding was one of the main forces driving the interaction between PTS and β-CD. These findings align with the stoichiometric ratio of the PTS/β-CD inclusion complexes deduced earlier through the solubility approach, which was 1:1.

### 2.4. Antioxidant Analysis of PTS/β-CD Inclusion Complexes Concentrations

Polyphenols are important secondary metabolites in plants and possess strong antioxidant activity [33]. Additionally, the intermolecular hydrogen bonding between β-CD and PTS may further enhance the antioxidant activity of the inclusion complexes [34]. The findings showed that DPPH and hydroxyl radical scavenging activities were positively correlated with the concentration of the PTS/β-CD inclusion complexes (Figure S1). At a concentration of 2.0 g/L, the PTS/β-CD complexes exhibited significantly higher DPPH and hydroxyl radical scavenging rates of 28.4 ± 1.7% and 33.2 ± 1.9%, respectively, compared to β-CD (*p* < 0.05). Compared to PTS, the PTS/β-CD complexes (2.0 g/L) exhibited significantly higher DPPH and hydroxyl radical scavenging rates of 33.3 ± 0.9% and 31.2 ± 0.4%, respectively, (*p* < 0.05).

### 2.5. Stability of PTS/β-CD Inclusion Complexes

The stability of PTS/β-CD inclusion complexes varied depending on different light conditions. As shown in Figure S2a, under both natural light and darkness, the retention rate of PTS in the inclusion complexes remained relatively unchanged over time (*p* > 0.05), indicating good stability of PTS under these conditions. However, after 40 h of exposure to UV light, only 51% of the PTS/β-CD inclusion complexes retained their initial value. This demonstrates that UV light significantly impacted the stability of PTS, suggesting that the inclusion complexes should be stored in conditions that avoid prolonged UV exposure. Additionally, while PTS/β-CD inclusion complexes remained stable under high humidity conditions, it showed a significant decrease in stability under high temperatures, with a 53.37% reduction after 12 h (Figure S2b).

### 2.6. Simulated Digestion of PTS/β-CD Inclusion Complexes In Vitro

As shown in Table S1 and Figure S3, although the total and reducing sugar concentrations of β-CD did not change significantly (*p* > 0.05) at certain stages of digestion, they decreased significantly when the digestion time increased (*p* < 0.05). The stability of β-CD was relative, and its cyclic structure could undergo local changes, resulting in some PTS detaching from the encapsulation of β-CD [35].

**Figure 11:**
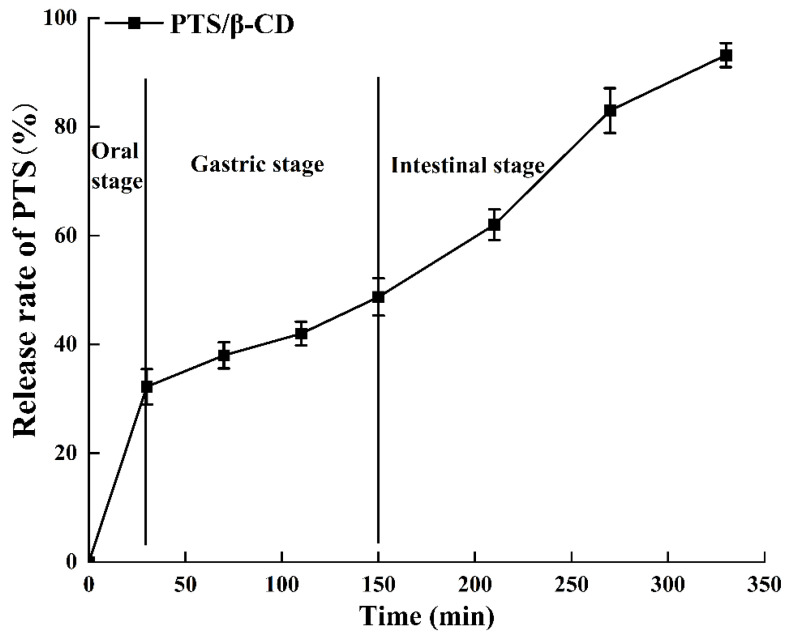
The release curve of PTS/β-CD inclusion complexes during simulated digestion in vitro.

As shown in Figure 11, PTS (32.22 ± 3.24%) was initially released in the oral cavity due to the degradation of the β-CD wall material by salivary enzymes, resulting in partial PTS release, which aligned with the changes in sugar content during oral digestion. The release curve during the gastric phase was stable with a low release rate, ending at 48.72 ± 3.42%. This low release rate may be due to the resistance of the PTS/β-CD inclusion complexes to the acidic conditions of the stomach. At the beginning of intestinal digestion, a rapid increase in PTS release was noticed, likely due to digestive enzymes in the simulated intestinal fluid breaking down the β-CD wall material. By the end of digestion, the final release rate of PTS reached 93.18 ± 2.22%. In summary, the encapsulation of PTS by β-CD allowed for targeted release in the intestine.

### 2.7. The Antioxidant Properties of PTS/β-CD Inclusion Complexes During Digestion in Vitro

The PTS/β-CD inclusion complexes’ antioxidant activity was studied using an in vitro digestion model (Figure S4). Both DPPH and hydroxyl free radical scavenging activities decreased significantly throughout the stages of digestion (*p* < 0.05). This reduction was likely due to changes in the digestive environment, which led to the rapid degradation of polyphenols and alterations in their structure, resulting in lower scavenging activity. However, as digestion progressed, the release of active substances exceeded the degradation rate, leading to a gradual increase in scavenging capacity [36]. In summary, the PTS/β-CD inclusion complexes retained significant antioxidant activity in the colon.

### 2.8. Influences of PTS/β-CD Inclusion Complexes on Intestinal Flora

#### 2.8.1. Influences of PTS/β-CD Inclusion Complexes on pH and Biomass of Intestinal Flora Fermentation

As shown in Figure S5a, the pH value in the blank group did not decrease significantly (*p* > 0.05). In contrast, the pH values of the FOS, β-CD, and PTS/β-CD inclusion complexes groups all decreased significantly at first (*p* < 0.05) and then stabilized. This trend was consistent with the changes observed in OD_600_ (Figure S5b). At the end of fermentation, the PTS/β-CD inclusion complexes group showed the greatest reduction in pH, reaching 4.54 ± 0.05. These results suggest that the fermentation of FOS, PTS, and PTS/β-CD may produce significant amounts of SCFAs, with probiotics exhibiting different substrate preferences. Increased SCFAs concentrations reduce the pH of the intestinal environment, which can inhibit the growth of pathogenic bacteria, such as *E. coli* [37]. However, as the culture period extended, the nutrients in the medium were mostly consumed, which slowed the growth of probiotics and reduced metabolic activity, causing the pH to stabilize.

#### 2.8.2. Influences of PTS/β-CD Inclusion Complexes on SCFAs of Intestinal Flora Fermentation

**Figure 12:**
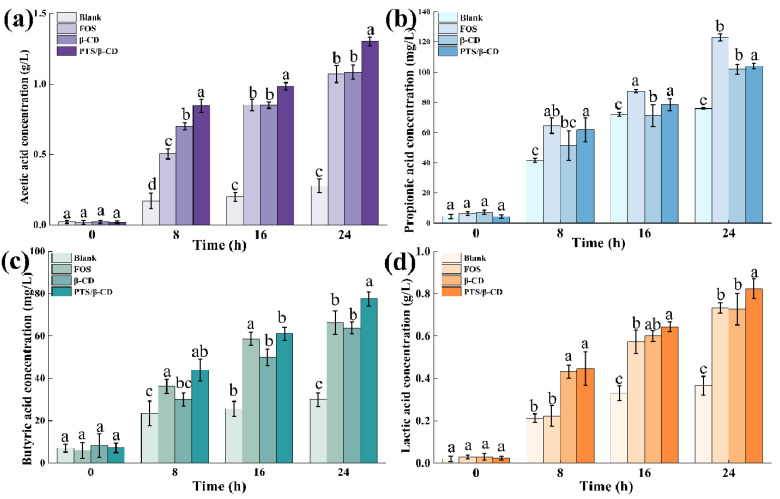
Effects of PTS/β-CD inclusion complexes during fecal fermentation on SCFAs concentrations in vitro. Acetic acid (**a**); Propionic acid (**b**); Butyric acid (**c**); Lactic acid (**d**). Note: Different letters indicated significant differences between groups at the same time (*p* < 0.05).

SCFAs are the main metabolites of the intestinal microbial community, which not only provide an important source of nutrients for intestinal epithelial cells but also play a key role in regulating the intestinal microenvironment, inhibiting inflammatory response, and enhancing the integrity of the intestinal barrier [38]. As the main short-chain fatty acid in the intestine, acetic acid can be absorbed by colon epithelial cells, participate in the metabolism of muscles, heart, brain, and other organs, and serve as their energy material [39]. As shown in Figure 12a, the acetic acid concentration produced by the PTS/β-CD inclusion complexes group (1.31 ± 0.03 g/L) was significantly higher than that of the FOS (1.07 ± 0.06 g/L) and β-CD groups (1.08 ± 0.05 g/L) (*p* < 0.05). Propionic acid has properties that inhibit cholesterol synthesis, reduce fat storage, and promote intestinal epithelial cell activity and hepatocyte autophagy [40,41]. The propionic acid contents in the β-CD and PTS/β-CD inclusion complexes groups were 101.83 ± 3.12 mg/L and 103.96 ± 1.75 mg/L, respectively, which were lower than the concentration in the FOS group (122.9 ± 2.42 mg/L) (Figure 12b).

Butyric acid helps maintain the stability of colorectal cells and can prevent or suppress cancer [42]. The butyric acid contents in the FOS, β-CD, and PTS/β-CD inclusion complexes groups were 66.22 ± 5.53 mg/L, 63.78 ± 2.77 mg/L, and 77.49 ± 3.27 mg/L, respectively. The production of butyric acid was notably elevated in the group treated with PTS/β-CD inclusion complexes, compared to both the FOS and β-CD groups (*p* < 0.05) (Figure 12c). Lactic acid is an important intermediate metabolite in the synthesis of SCFAs by intestinal flora using prebiotics and can be used as substrate to directly produce propionic acid through acrylate pathway [43]. The lactic acid concentration in the PTS/β-CD inclusion complexes group was 0.82 ± 0.04 g/L (Figure 12d).

These results suggest that the FOS, β-CD, and PTS/β-CD inclusion complexes groups increased SCFA production compared to the blank group. Additionally, differences in SCFA yield and composition between the β-CD and PTS/β-CD inclusion groups could be attributed to structural differences, leading to distinct degradation pathways during static fermentation.

#### 2.8.3. Effects of PTS/β-CD Inclusion Complexes on Intestinal Flora

**Figure 13:**
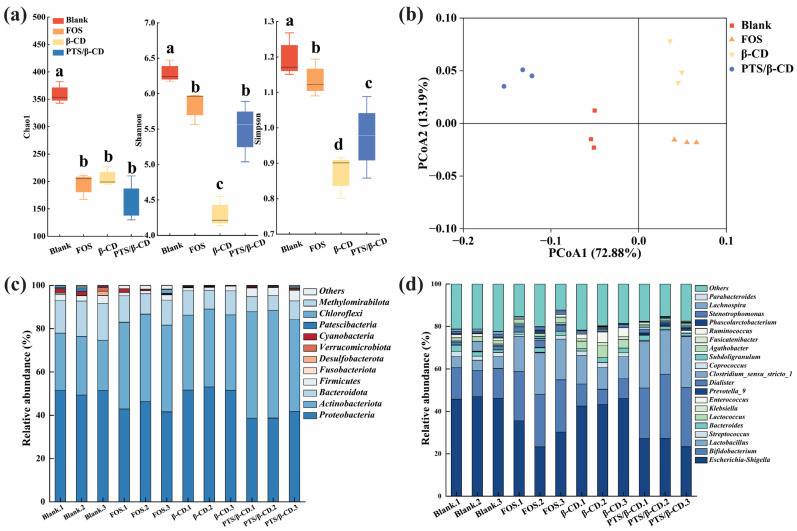
Effects of different PTS/β-CD inclusion complexes on gut microbiota composition of healthy human. The α diversity of gut microbiota (**a**); principal coordinates analysis of microbiota at operational taxonomic units (OUTs) levels based on weighted Unifrac distances (**b**); and bar plot of prevalence at the phylum (**c**) and genus (**d**) levels. Note: Different letters in Figure (**a**) indicated significant differences between samples (*p* < 0.05).

Based on the comparison of bacterial richness (Chao1 index) and diversity (Shannon and Simpson index), the Chao1 index of the PTS/β-CD group decreased slightly compared to the β-CD group. However, the Simpson and Shannon indexes were significantly higher than those in the β-CD group (Figure 13a). Therefore, the PTS/β-CD group significantly increased the diversity of gut microbes, which may be closely related to the release and metabolism of polyphenols (*p* < 0.05) [44]. Based on the weighted UniFrac distance, principal coordinates analysis (PCoA) was performed, revealing that the microflora structure of each group clustered distinctly, showing significant differences (Figure 13b) [37]. The results indicated that the gut microflora responded differently to various carbon sources. However, since the role of gut microbes in metabolism was complex, further investigation at the phylum and genus levels was required to explore the potential effects of PTS/β-CD released polyphenols on microbial composition.

At the phylum level, *Proteobacteria*, *Firmicutes*, *Actinobacteria*, and *Bacteroidetes* accounted for more than 90% of the total microflora in all groups (Figure 13c). These results align with the typical composition and structure of human gut flora, suggesting that fecal fermentation tests in vitro could effectively simulate human gut microbiota [45]. Compared to the β-CD group, the relative abundance of *Actinobacteria* and *Firmicutes* increased in the PTS/β-CD group, while the relative abundance of *Proteobacteria* decreased significantly (*p* < 0.05). *Actinobacteria* were primarily represented by *Bifidobacterium*, and most members of *Firmicutes* were beneficial bacteria, such as *Lactobacillus*. In contrast, many bacteria in *Proteobacteria* are pathogenic to humans, such as *Escherichia coli* and *Klebsiella*. After 24 h of fermentation, the relative abundance of *Bacteroides* in the FOS, β-CD, and PTS/β-CD groups were lower than in the blank group, which may be related to the lower pH value of the fermentation environment. Previous studies [46] have shown that acidic conditions inhibit the growth of acid-sensitive *Bacteroides*, leading to a decrease in Bacteroidetes abundance after fermentation.

At the genus level (Figure 13d), compared to the FOS and β-CD groups, the PTS/β-CD inclusion complexes group showed a significant increase in the number of probiotic genera, such as *Bifidobacterium* and *Lactobacillus* (*p* < 0.05), while the growth of opportunistic pathogens, like *Lactococcus*, *Streptococcus*, and *Klebsiella*, was significantly inhibited (*p* < 0.05) after 24 h of fermentation. Additionally, in the β-CD group, *Lactobacillus* increased and *Klebsiella* decreased compared to the blank group. In summary, β-CD provides mutual benefits when encapsulating PTS. It not only serves as an effective embedding carrier but also acts as a prebiotic, influencing the abundance and composition of intestinal flora. The inclusion of PTS in the PTS/β-CD group further affects the gut microbiota’s composition and abundance, while promoting positive effects on gut barrier function.

## 3. Materials and Methods

### 3.1. Materials and Chemicals

PTS (Purity ≥97%) was purchased from Guangzhou Kereng Biotechnology Co., Ltd. (Guangzhou, China) β-CD and ethanol were obtained from Sinopharm Group Chemical Reagent Co., Ltd. (Beijing, China). Artificial gastric juice and artificial intestinal juice were sourced from Nanjing Xinfan Biotechnology Co., Ltd. (Jiangsu, China). Thin layer chromatography (TLC) plates were acquired from Green Baicao Technology Co., Ltd. (Shenzen, China). All remaining reagents were domestic analytical grade.

Static anaerobic basal fermentation medium (g/L): carbon source 0.5; tryptone 0.2; yeast extract 0.2; NaCl 0.01; K_2_HPO_4_ 0.004; KH_2_PO_4_ 0.04; MgSO_4_·7H_2_O 0.001; CaCl_2_·6H_2_O 0.001; NaHCO_3_ 0.2; heme chloride 0.0025; vitamin K_1_ (V_K1_) 0.002 (sterilized by filtration); L-cysteine hydrochloride 0.05; bile salt 0.05, Azurazine solution (0.25 g/L) 0.4 mL, pH 6.8–7.0.

### 3.2. Experimental Method

#### 3.2.1. Phase Solubility Curve

The phase solubility investigation was conducted using the technique outlined by Higuchi and Connors [46]. Aqueous solutions (50 mL) of 0.0, 1.0, 1.5, 2.0, 2.5, and 3.0 mmol/L of β-CD, α-cyclodextrin (α-CD), and 2-hydroxypropyl-β-cyclodextrin (2-HD-β-CD) were prepared, and excess PTS (5.0 mmol/L; 50 mL) was added to each solution, followed by magnetic stirring for 72 h at 30 ± 2 °C and 1000 r/min, while avoiding light. Three parallel samples were established for each set of solutions to minimize experimental errors. After filtration, the PTS concentrations in the solution were measured utilizing the UV approach (306 nm), and phase solubility curves for PTS with the different CDs were plotted. A linear regression analysis of CD concentration (X) versus PTS concentration (Y) was performed to calculate the stability constant (*Kc*) of the inclusion complexes using Formula (1):

(1) $$\mathrm{Kc}\;(M^{-1})=\frac{\mathrm{slope}}{S_{0\;}\left(1-\mathrm{slope}\right)}$$

where S_0_ represented the PTS concentration in the absence of host molecules (54 μM at 23 °C) [47].

#### 3.2.2. Synthesis of PTS/β-CD Inclusion Complexes

PTS/β-CD inclusion complexes were synthesized employing the co-evaporation and lyophilization procedures. β-CD (0.908 g) and PTS (0.205 g) were precisely measured in a 1:1 molar proportion and dissolved in 10% ethanol solution (0.4 L). The mixture was agitated in the dark under 30 °C and 1000 r/min for 48 h. Finally, the ethanol solvent was evaporated and filtered (0.45 μm). The resulting product was then vacuum freeze-dried.

#### 3.2.3. Preparation of PTS/β-CD Physical Mixtures

The physical inclusion complexes were prepared with a 1:1 molar ratio of β-CD to PTS. The two compounds were mixed in a quartz crucible and ground for 15 min to produce the PTS/β-CD physical inclusion complexes.

#### 3.2.4. Analysis of PTS/β-CD Inclusion Complexes

Samples of PTS, β-CD, the PTS/β-CD physical mixture, and the PTS/β-CD inclusion complexes were mounted on the sample table, coated with platinum, and observed and photographed at 5.0 kV under magnifications of 2.5Kx (Hitachi S-4800, Hitachi, Hitachi City, Japan).

The samples prepared using the optimal process were analyzed. The infrared spectra were measured using the KBr tablet method (Shimadzu IRPrestige-21, Shimadzu, Kyoto, Japan). The spectra were scanned over a wavelength range of 4000–400 cm^−1^.

Samples (5.0 mg) were placed in an aluminum crucible for analysis using a differential scanning calorimeter (Shimadzu DSC-60A, Shimadzu, Kyoto, Japan). The measurement parameters were set as follows: nitrogen flow rate of 40 mL/min, heating rate of 10 °C/min, and scanning temperature range of 50–300 °C.

The β-CD, PTS, PTS/β-CD inclusion complexes, and PTS/β-CD physical inclusion complexes were analyzed using X-ray diffraction (SmartLab SE, Kyoto, Japan). A diffractometer with a Cu Kα anode (λ = 1.54060 Å) operating at 40 kV and 50 mA was used. The test conditions were set as follows: diffraction angle 2θ ranging from 10–100°, scanning rate of 15°/min, and the test was conducted at room temperature (25 ± 2 °C).

The β-CD was dissolved in D_2_O, while PTS was dissolved separately in DMSO-d6. To prepare the PTS/β-CD inclusion complexes (10.0 mg), they were dissolved in a solution containing 80% D_2_O. All samples were then analyzed using the ^1^H NMR spectrometer (Bruker AV 500M, Bruker Corporation, Berlin, Germany).

#### 3.2.5. Molecular Docking

The receptor (β-CD: 444041) and the ligand (PTS: 5281727) were downloaded as 3D models from the PubChem database (https://pubchem.ncbi.nlm.nih.gov/) (accessed on 7 November 2024). ChemDraw Ultra (Version 14.0) was then used to minimize energy and optimize the models. Following this, AutoDock-Tools 1.5.6 was employed to process the receptors and ligands by adding hydrogen atoms, assigning atomic charges, setting the number of single bonds, and defining the root atoms in PTS molecules. The processed receptors and ligands were saved in pdbqt format. Subsequently, AutoDock-Vina was used for semi-flexible molecular docking, treating the entire β-CD structure as a potential binding site. For docking coordinates, the conformation with the lowest free binding energy was selected, and the results were visualized and analyzed using Pymol software (Version 3.1.0).

#### 3.2.6. Different Concentration of PTS/β-CD Inclusion Complexes Antioxidant Activity

According to the methods of Yang et al., the scavenging abilities of the samples against the 2,2-diphenyl-1-picrylhydrazyl (DPPH) radical scavenging assay and hydroxyl free radicals were measured [48]. Vitamin C (Vc) was used as a positive control.

#### 3.2.7. Stability Experiment of PTS/β-CD Inclusion Complexes

To assess the effect of light on the stability of PTS/β-CD inclusion complexes, the samples (4 mg/L) were dissolved in ultrapure water (4 mg/L) and, subsequently, exposed to natural light, ultraviolet light, and darkness for 200 h. The absorbance of PTS/β-CD inclusion complexes was measured every 5 h to monitor any changes.

The aqueous solution of PTS/β-CD inclusion complexes (4 mg/L) was exposed to high temperature (60 ± 2 °C) and high humidity (60 ± 2%). Absorbance measurements were taken every 2 d to assess the stability of the complexes under these conditions.

#### 3.2.8. Simulated Digestion of PTS/β-CD Inclusion Complexes in Saliva, Gastric Juice, and Small Intestine In Vitro

An amount of 40 mL of artificial saliva was added to 40 mL of sample solution (PTS/β-CD, β-CD, and ultrapure water). The salivary digestive fluid was placed in a shaker incubator (37 °C, 100 r/min) for in vitro simulated digestion. Then, 2 mL samples were taken at 0, 10, 20, and 30 min and boiled (10 min) to inactivate salivary amylase. The leftover digestive fluid was, subsequently, employed to simulate gastric fluid digestion.

The remaining salivary digestive fluid was mixed with the artificial gastric fluid at the rate of 1:1 (*v*:*v*). Samples (2 mL) were gathered under 0, 40, 80, and 120 min, and each sample was boiled (10 min). The remaining gastric digestive fluid was then combined with artificial intestinal fluid at a 10:3 ratio (*v*:*v*) and placed in a shaking incubator for further digestion. Small intestinal fluid samples (2 mL) were taken at 0, 1, 2, and 3 h, boiled, and subsequently, analyzed. The total sugar and reducing sugar contents were determined using the anthrone–sulfuric acid and DNS colorimetric methods, respectively. Additionally, the antioxidant activity of the samples at different digestion stages was measured [48].

According to Wu et al., TLC was employed to monitor the simulated digestion process of PTS/β-CD inclusion complexes [49]. Then, the concentrations of free PTS were determined by high-performance liquid chromatography (HPLC) (Waters E2695, Waters, Milford, MA, USA) with C18 column (4.6  × 250 mm) with the following parameters: gradient elution: 0–12 min, 10–20% A; 12–20 min, 20–28% A; 20–35 min, 28–40% A; 35–40 min, 40% A, where solvent A was acetonitrile, and solvent B was 0.1% phosphoric acid; detection wavelength, 306 nm; injection volume, 20 μL; flow rate, 1 mL/min; detection temperature, 30 °C; regression equation, Y  =  18.229X − 2.8; correlation coefficient, 0.9996; range (mM/L): 0.0–5.0. The release rate of PTS from the PTS/β-CD inclusion complexes at each stage of the simulated digestion was calculated using Formula (2):

(2) $$\mathrm{Release}\;\mathrm{rate}\;\mathrm{of}\;\mathrm{PTS}\;=\frac{Free\;PTS\;(\mathrm{mg})}{Embedded\;PTS\;(\mathrm{mg})}\times 100\%$$

#### 3.2.9. Fecal Flora Fermentation in Healthy Human In Vitro

Fecal samples from four healthy volunteers (two males and two females, aged 20–25 years) were collected through sterile containers. Under anaerobic conditions, equal quantities of the fresh samples were blended and, subsequently, diluted with sterile PBS to create a fecal homogenate of 10 g/L. The mixture was filtered through eight layers of gauze under aseptic conditions to remove any residue. The fecal homogenate was then inoculated into different groups of intestinal anaerobic basal nutrient media. The negative control group (blank group) consisted of the intestinal anaerobic basal nutrient medium without any carbon source, while the experimental groups were supplemented with FOS (positive control group), β-CD, and PTS/β-CD (5 g/L) as carbon sources. The samples were incubated under 37 °C (24 h), with aliquots gathered under 0, 8, 16, and 24 h for subsequent analysis. The total sugar and reducing sugar contents were determined using the anthrone–sulfuric acid method and DNS colorimetric method, respectively. Additionally, the fermentation pH and absorbance values (OD_600_) were measured.

#### 3.2.10. Measurement of SCFAs and Lactic Acid Concentrations

After centrifuging the fermentation samples at various stages (12,000 r/min for 5 min), 100 μL of the supernatant was collected and diluted 10 times with 60% ethanol. An amount of 2-ethylbutyric acid, at a final concentration of 1 g/L, was added as the internal standard. The mixture was oscillated and mixed thoroughly, and 300 μL of the prepared sample was analyzed using a gas chromatograph (Shimadzu NexisGC-2030, Shimadzu, Kyoto, Japan) to determine the content of SCFAs. Lactic acid concentrations were measured using a biosensor analyzer (SBA-40ES, Institute of Biology, Shandong Academy of Sciences, Jinan City, China).

#### 3.2.11. Detection of Fecal Flora Composition

Genomic DNA was extracted using the CTAB/SDS method and diluted to a concentration of 1 ng/μL. Specific primers were selected based on the diluted genomic DNA, and the V_3_–V_4_ regions of the 16_S_ rRNA gene were amplified by PCR. The resulting PCR products were then purified. Libraries for sequencing were prepared using the NEBNext^®^ Ultra™ II DNA Library Prep Kit, and their quantities were assessed through the Qubit and Q-PCR methods. Subsequently, the sequencing was performed on the Illumina NovaSeq platform.

#### 3.2.12. Statistical Analysis

Data mapping was performed using Origin 2022 software, with all experiments conducted in three biological replicates. Statistical analysis was carried out using one-way ANOVA followed by Duncan’s test (Mean ± SD, *n* = 3), and SPSS 19.0 software was used for further evaluation. Different letters were used to indicate significant differences (*p* < 0.05).

## 4. Conclusions

In this research, the preparation of PTS/β-CD inclusion complexes was successfully achieved through the combination of stirring and freeze-drying, and their structural characteristics were analyzed through FT-IR, XRD, DSC, and SEM techniques. Phase solubility experiments indicated that β-CD could theoretically form 1:1 inclusion complexes with PTS. Antioxidant studies demonstrated that PTS/β-CD inclusion complexes significantly enhanced the scavenging ability of PTS on hydroxyl radicals and DPPH radicals. In vitro digestion experiments revealed that β-CD exhibited resistance to digestion in simulated saliva, gastric fluid, and small intestinal fluid, allowing it to reach the colon intact. During simulated gastrointestinal digestion, PTS in the PTS/β-CD inclusion complexes was gradually released, with a final release rate of 93.18 ± 2.22%. In vitro probiotic experiments showed that, compared to the β-CD and FOS groups, PTS/β-CD significantly increased the production of acetic acid, butyric acid, and lactic acid within 24 h, facilitated the proliferation of beneficial bacteria, such as *Bifidobacterium* and *Lactobacillus*, and inhibited the proliferation of potential pathogens, such as *Lactococcus*, *Streptococcus*, and *Klebsiella*. Additionally, in comparison to the blank group, the propionic and butyric acids contents in the β-CD group were significantly higher. The abundance of key beneficial bacteria, like *Lactobacillus*, increased, while there was a marked decrease in the relative abundance of pathogenic bacteria like *Klebsiella*. In conclusion, PTS/β-CD inclusion complexes modulated the gut microbiota composition, fostering the proliferation of beneficial bacteria and suppressing harmful bacteria, resulting in dual probiotic functions. However, the application of PTS/β-CD in the food industry remains in its early stages, and future research should focus on cell and animal studies to provide more robust data and theoretical support for its use in food products.

## Data Availability

Data will be made available on request.

## Supplementary Materials

The following supporting information can be downloaded at: https://www.mdpi.com/article/10.3390/molecules30061363/s1, Table S1. Changes of the total and reducing sugar contents in PTS/β-CD inclusion complexes. Figure S1 The antioxidant capacity activity of different concentrations of PTS/β-CD inclusion 22 complexes. DPPH radical scavenging activity (a); hydroxyl radical scavenging activity (b). Figure S2 Stability of PTS/β-CD inclusion complexes. (a): Effect of natural UV light, light, and darkness 24 on the stability of PTS/β-CD inclusion complexes; (b): Effect of high humidity and high temperature 25 on the stability of PTS/β-CD inclusion complexes. Figure S3 Effects of simulated digestion on PTS/β-CD inclusion complexes by TLC in vitro. Figure S4 Changes in DPPH radical (a) and hydroxyl radical (b) of PTS/β-CD inclusion complexes 28 during simulated digestion. Note: Different letters indicate significant differences at different time points within the same 30 digestion interval (*p* < 0.05). Figure S5 Results of the effects of PTS/β-CD inclusion complexes on the pH and growth of medium: pH 32 (a); OD600 (b).
