# Supplementary material for: Characterization and In Vitro Prebiotic Activity of Pterostilbene/β-Cyclodextrin Inclusion Complexes
Source: Molecules. 2025 Mar 18;30(6):1363. doi: 10.3390/molecules30061363 (PMC11944276; doi:10.3390/molecules30061363)
Supplement: Supplementary file 1 [file molecules-30-01363-s001.zip › molecules-3494297-supplementary.pdf]

Supplementary Information

Characterization and *in vitro* prebiotic activity of pterostilbene/β-cyclodextrin inclusion complexes

Chuan-Chao Wu[ab], Long Qian[a], Zhen Rong[a], Yu-Qi Li[a], Hai-Xia Wu[c], Hui-Min Zhang[a], Rui-Yu He[ad*], Guo-Qiang Zhang[ab*].

[a] College of Biology and Food Engineering, Anhui Polytechnic University, 241000, Wuhu, China.

[b] Wuhu Green Food Industrial Research Institute Co., LTD, 241000, Wuhu, China.

[c] Department of orthopedics, Lianshui County People's Hospital, Huaian, 223001, China

[d] School of Biotechnology, Jiangnan University, 214000, Wuxi, China.

Corresponding author:

Rui-Yu He*, Guo-Qiang Zhang*

Email: 6240207007@stu.jiangnan.edu.cn; zhangguoqiang@ahpu.edu.cn

Captions of Table and Figures

Table S1. Changes of the total and reducing sugar contents in PTS/β-CD inclusion complexes.

Fig. S1 The antioxidant capacity activity of different concentrations of PTS/β-CD inclusion complexes. DPPH radical scavenging activity (a); hydroxyl radical scavenging activity (b).

Fig. S2 Stability of PTS/β-CD inclusion complexes. (a): Effect of natural UV light, light, and darkness on the stability of PTS/β-CD inclusion complexes; (b): Effect of high humidity and high temperature on the stability of PTS/β-CD inclusion complexes.

Fig. S3 Effects of simulated digestion on PTS/β-CD inclusion complexes by TLC *in vitro*.

Fig. S4 Changes in DPPH radical (a) and hydroxyl radical (b) of PTS/β-CD inclusion complexes during simulated digestion.

Note: Different letters indicate significant differences at different time points within the same digestion interval (P<0.05).

Fig. S5 Results of the effects of PTS/β-CD inclusion complexes on the pH and growth of medium: pH (a); OD_{600} (b).

Table S1.

	Times (min)	Total sugar content (g/L)*	Reducing sugar content (g/L)*
Salivary	0	1.02±0.007^{a}	0.014±0.007^{a}
	10	1.05±0.014^{a}	0.014±0.004^{a}
	20	0.93±0.010^{a}	0.013±0.005^{a}
	30	1.01±0.005^{a}	0.011±0.011^{a}
Gastric	0	0.95±0.016^{a}	0.027±0.018^{a}
	40	0.93±0.026^{a}	0.030±0.028^{a}
	80	0.89±0.014^{a}	0.034±0.011^{a}
	120	0.88±0.019^{a}	0.031±0.010^{a}
Small intestinal	0	0.80±0.019^{a}	0.040±0.016^{a}
	60	0.78±0.012^{a}	0.042±0.005^{a}
	120	0.76±0.003^{a}	0.046±0.021^{a}
	180	0.75±0.033^{a}	0.052±0.020^{a}

* Different superscript letters indicated significant differences (P < 0.05) between time points in the same compartment.

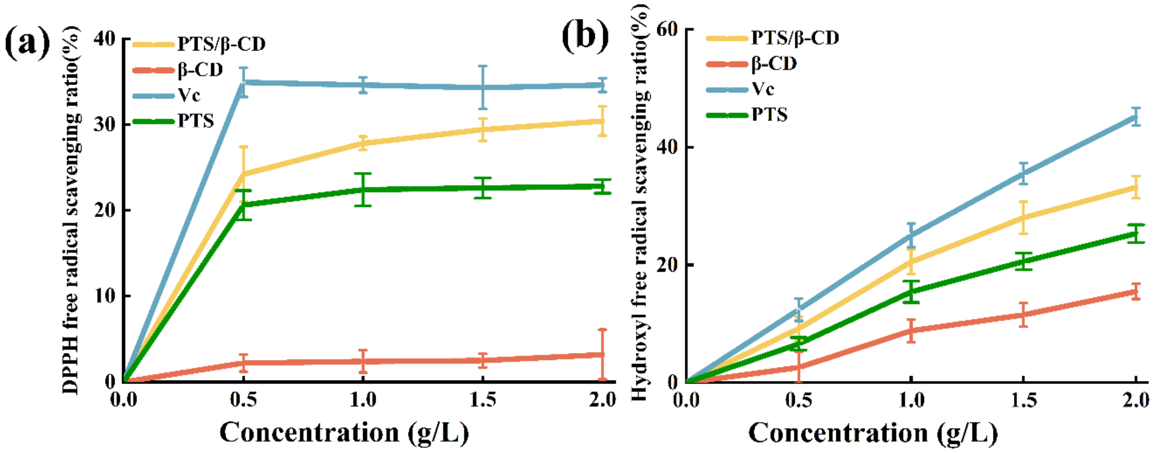
(a)
PTS/β-CD
β-CD
Vc
PTS
DPPH free radical scavenging ratio(%)
Concentration (g/L)
(b)
Hydroxyl free radical scavenging ratio(%)
Concentration (g/L)

Fig. S1

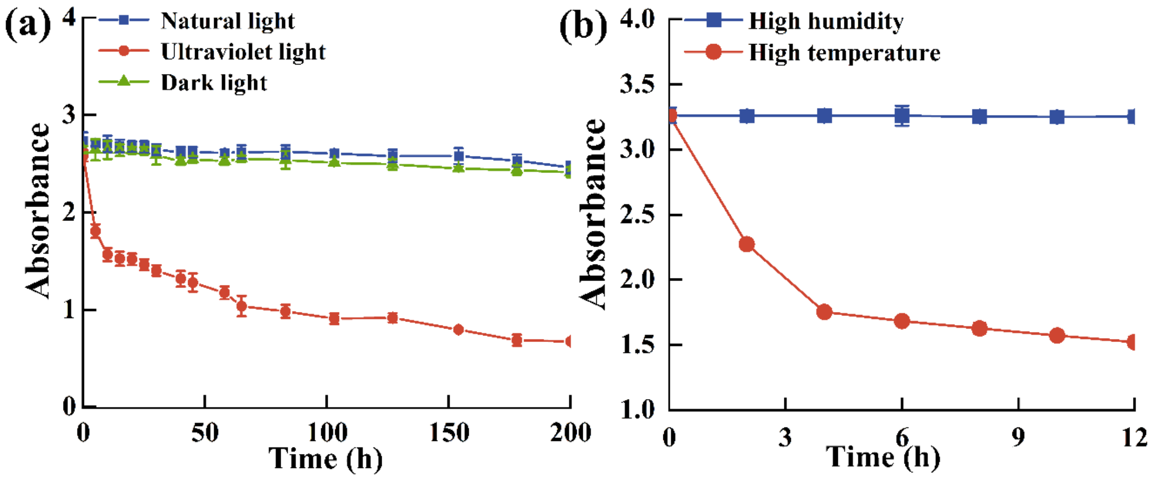
(a)
Natural light
Ultraviolet light
Dark light
Absorbance
Time (h)
(b)
High humidity
High temperature
Absorbance
Time (h)

Fig. S2

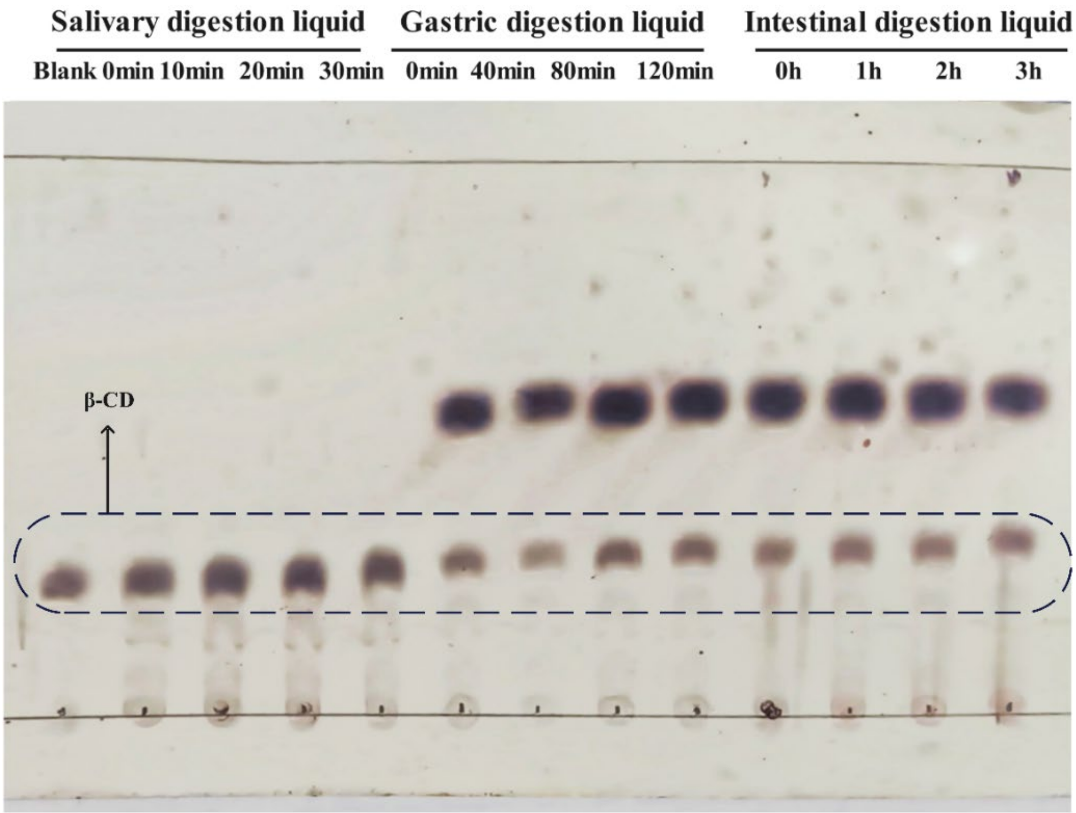
Salivary digestion liquid
Gastric digestion liquid
Intestinal digestion liquid
Blank
0min
10min
20min
30min
0min
40min
80min
120min
0h
1h
2h
3h
β-CD

Fig. S3

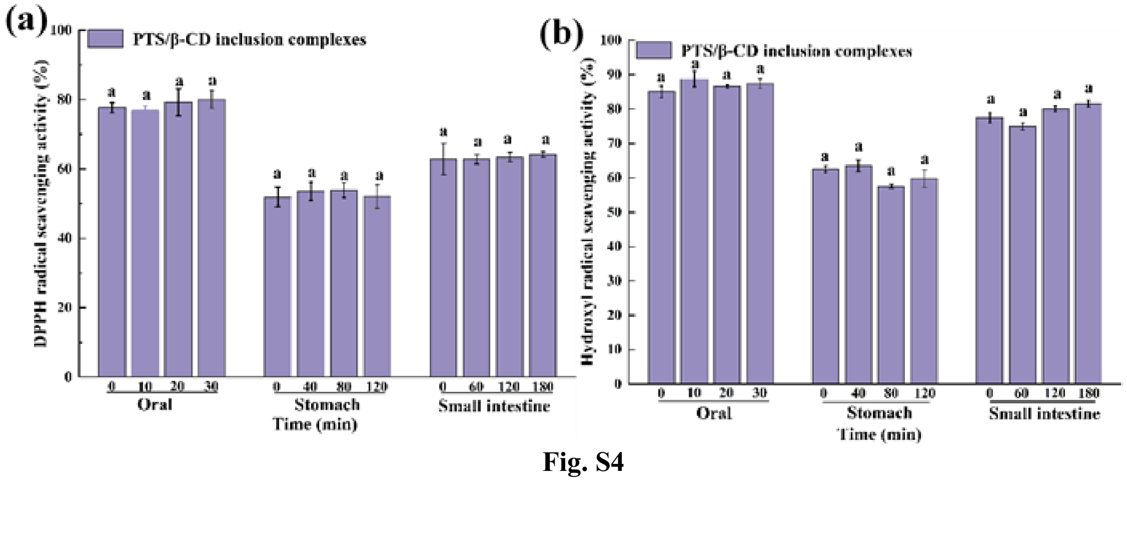
(a)
PTS/β-CD inclusion complexes
DPPH radical scavenging activity (%)
Oral
Stomach
Small intestine
Time (min)
(b)
PTS/β-CD inclusion complexes
Hydroxyl radical scavenging activity (%)
Oral
Stomach
Small intestine
Time (min)

Fig. S4

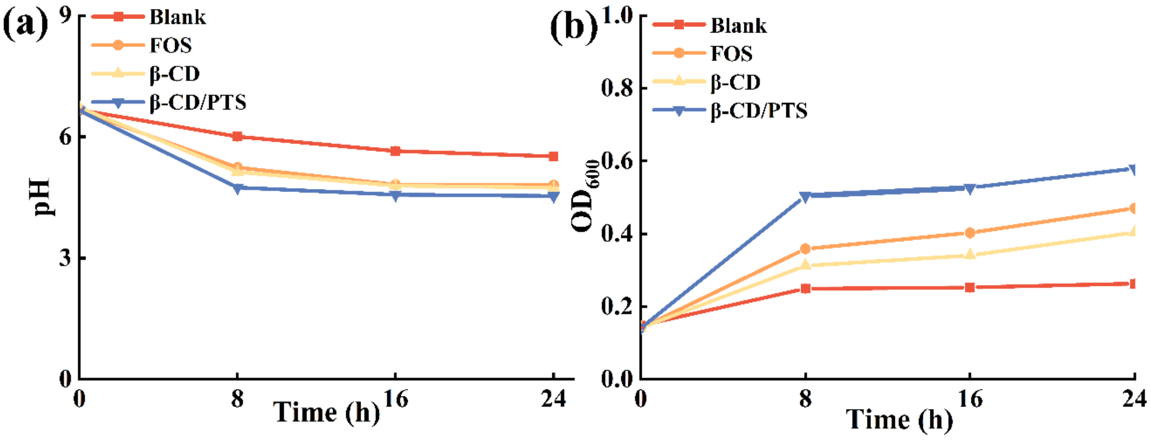
(a)
Blank
FOS
β-CD
β-CD/PTS
pH
Time (h)
(b)
Blank
FOS
β-CD
β-CD/PTS
OD600
Time (h)

Fig. S5
